# The relation between delayed reperfusion treatment and reduced left ventricular ejection fraction in patients with ST-segment elevation myocardial infarction: a national prospective cohort study

**DOI:** 10.1093/ehjopen/oeaf034

**Published:** 2025-04-02

**Authors:** Bård Uleberg, Kaare Harald Bønaa, Kari Krizak Halle, Bjarne K Jacobsen, Beate Hauglann, Eva Stensland, Olav Helge Førde

**Affiliations:** Department of Community Medicine, UiT The Arctic University of Norway, Postboks 6050 Stakkevollan, 9037 Tromsø, Norway; Centre for Clinical Documentation and Evaluation (SKDE), Northern Norway Regional Health Authority, Postboks 6400, 9294 Tromsø, Norway; Faculty of Medicine, Department of Circulation and Medical Imaging, Norwegian University of Science and Technology, Postboks 8905, 7491 Trondheim, Norway; Clinic for Heart Disease, St. Olavs University Hospital, Postboks 3250, Torgarden, 7006 Trondheim, Norway; Department of Medical Quality Registers, St. Olavs University Hospital, Postboks 3250, Torgarden, 7006 Trondheim, Norway; Department of Medical Quality Registers, St. Olavs University Hospital, Postboks 3250, Torgarden, 7006 Trondheim, Norway; Department of Community Medicine, UiT The Arctic University of Norway, Postboks 6050 Stakkevollan, 9037 Tromsø, Norway; Centre for Clinical Documentation and Evaluation (SKDE), Northern Norway Regional Health Authority, Postboks 6400, 9294 Tromsø, Norway; Department of Community Medicine, Centre for Sami Health Research, UiT The Arctic University of Norway, Postboks 6050, Langnes, 9037 Tromsø, Norway; Centre for Clinical Documentation and Evaluation (SKDE), Northern Norway Regional Health Authority, Postboks 6400, 9294 Tromsø, Norway; Department of Community Medicine, UiT The Arctic University of Norway, Postboks 6050 Stakkevollan, 9037 Tromsø, Norway; Centre for Clinical Documentation and Evaluation (SKDE), Northern Norway Regional Health Authority, Postboks 6400, 9294 Tromsø, Norway; Department of Community Medicine, UiT The Arctic University of Norway, Postboks 6050 Stakkevollan, 9037 Tromsø, Norway; Centre for Clinical Documentation and Evaluation (SKDE), Northern Norway Regional Health Authority, Postboks 6400, 9294 Tromsø, Norway

**Keywords:** Acute myocardial infarction (AMI), PCI, Fibrinolysis

## Abstract

**Aims:**

The aim of this nationwide study of patients with acute ST-segment elevation myocardial infarction (STEMI) was to investigate the relation between delayed reperfusion and mildly to moderately reduced and severely reduced left ventricular ejection fraction (LVEF).

**Methods and results:**

In this national population-based cohort study, log-binominal and modified Poisson regression models were applied to examine the associations between delayed reperfusion (i.e. fibrinolysis > 30 min or primary percutaneous coronary intervention > 120 min after first medical contact) and reduced LVEF, adjusted for reperfusion strategy, and patient characteristics. A total of 6567 Norwegian patients with STEMI registered in the Norwegian Registry of Myocardial Infarction during 2015–2018 were included in the analyses. Among them, 57% had normal LVEF (≥50%), 39% had mildly to moderately reduced LVEF (31–49%), and 4% had severely reduced LVEF (≤30%), measured during the acute admission. The adjusted relative risk of having a mildly to moderately reduced LVEF was 1.11 [95% confidence interval (CI) 1.04–1.18] for patients receiving delayed vs. timely reperfusion, and the adjusted relative risk of having severely reduced LVEF was 1.76 (95% CI 1.37–2.25) for patients receiving delayed vs. timely reperfusion. Reperfusion strategy, either primary percutaneous coronary intervention (pPCI) or a pharmacoinvasive strategy (PI), was not a significant determinant for reduced LVEF in any of the analyses.

**Conclusion:**

Delayed reperfusion treatment in STEMI increases the risk of mildly to moderately reduced LVEF, and the risk of severely reduced LVEF substantially, compared with timely reperfusion. The risk of reduced LVEF did not differ between patients treated with pPCI or PI.

## Graphical Abstract

**Graphical Abstract:**
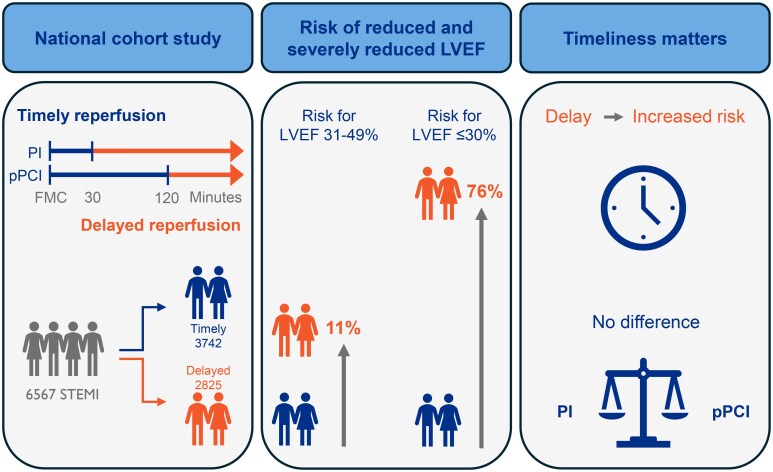

## Introduction

Early establishment of diagnosis and subsequent reperfusion of the occluded coronary artery in patients with ST-segment elevation myocardial infarction (STEMI) are crucial to avoid myocardial damage and to reduce the risk of heart failure and death.^1^ To ensure timely reperfusion for patients with STEMI, the European Society of Cardiology (ESC) recommends primary percutaneous coronary intervention (pPCI), provided that wire crossing of the infarct-related artery can be performed within 120 min of diagnosis. If this time recommendation cannot be met, a pharmacoinvasive strategy (PI) is recommended with administration of fibrinolytic within 10 min from STEMI diagnosis in patients without contraindications, followed by a coronary angiography and a rescue PCI if indicated.^1^ The corresponding recommendations in Norway are pPCI, defined as arterial access, within 120 min from first medical contact (FMC), or, if this time limit cannot be met, PI within 30 min of FMC.^2^

The Norwegian national goal for STEMI treatment is that at least 85% of the patients should receive timely reperfusion. However, according to the Norwegian Registry of Myocardial Infarction (NORMI), only 56% of the patients with STEMI received timely reperfusion during 2015–2018,^3^ increasing to 69% in 2023.^2^ A Norwegian study based on nationwide data on all patients with STEMI admitted to hospital during 2013–2019 showed that receiving timely reperfusion by the PI strategy was associated with better survival compared with delayed or late pPCI.^4^

ST-segment elevation myocardial infarction is frequently complicated with reduced left ventricular ejection fraction (LVEF) and heart failure. Studies have reported higher LVEF after pPCI than after fibrinolysis.^5,6^ None of these studies, however, compared patients receiving delayed reperfusion to patients receiving timely reperfusion. The aim of this study was to investigate the relationships between delayed reperfusion and mildly to moderately reduced LVEF (31–49%) and severely reduced LVEF (≤30%), by using data from a national medical quality register and patient administrative registries.

## Materials, methods, and study population

### Data sources and study design

**Figure 1:**
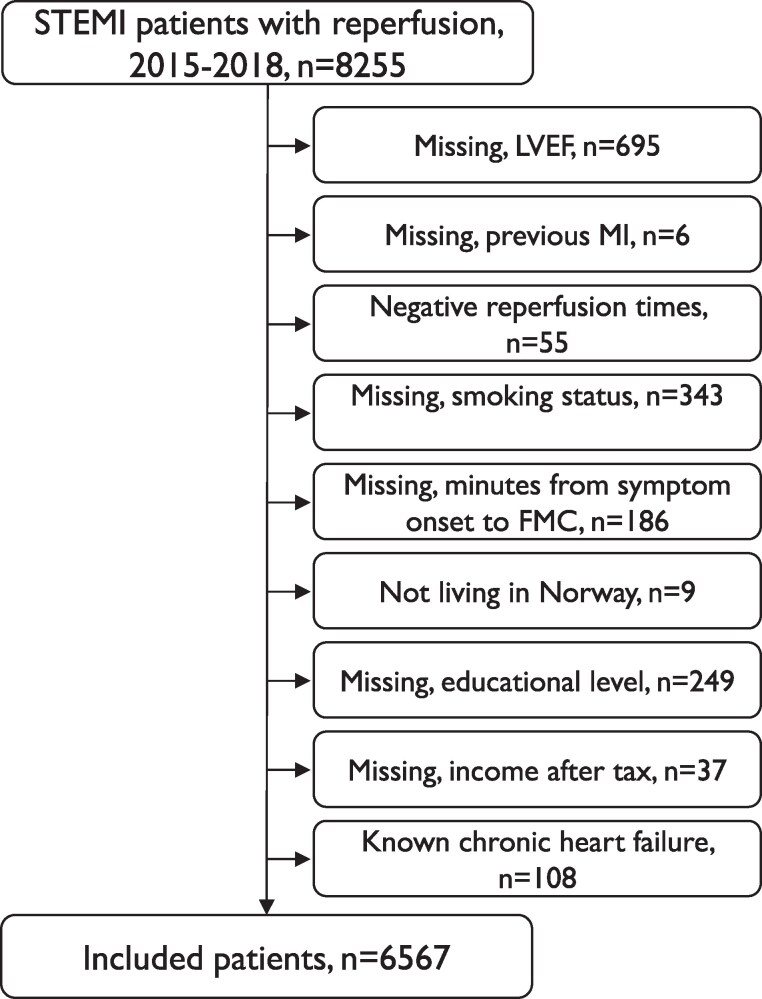
Patients with ST-segment elevation myocardial infarction and reperfusion treatment, 2015–2018. FMC, first medical contact; LVEF, left ventricular ejection fraction; STEMI, ST-segment elevation myocardial infarction.

During 2015–2018, 8255 Norwegian patients with STEMI younger than 85 years of age received reperfusion treatment within 12 h from symptom onset to FMC and were registered in the NORMI. For this study, we excluded patients with known chronic heart failure, patients with negative reperfusion times, patients not living in Norway, and patients with missing values for included covariates (*Figure 1*), and the study population accordingly consists of 6567 patients.

The NORMI is a national clinical quality register within the Norwegian Cardiovascular Disease Register (NCDR). Reporting to NCDR and NORMI is mandatory for Norwegian hospitals and does not require patient consent. The main purpose of the NORMI is to support the improvement in quality of care for acute myocardial infarction. The register contains data on medical history and risk factors, symptoms, treatments, and complications. An encrypted patient identification number, derived from the unique 11-digit Norwegian national identity number, allowed us to supplement data from other national registers by linkage. Information on all specialized healthcare services during 2013–2018 was obtained from the Norwegian Patient Register (NPR) and used to compose the comorbidity index included in the analyses. Individual data for the patients’ educational level and income after tax were obtained from Statistics Norway. All other variables included in the analysis were obtained from the NORMI.

### Definitions

The outcome of this study was LVEF following reperfusion treatment in patients with STEMI, measured during the acute admission. The latest measurement was used in the case of several measurements during hospitalization. ST-segment elevation myocardial infarction was defined according to the fourth universal definition of myocardial infarction.^7^ In 2015 and 2016, the NORMI did not distinguish between mildly and moderately reduced LVEF. For this study, LVEF was therefore categorized into normal (≥50%), mildly to moderately reduced (31–49%), and severely reduced (≤30%).

The reperfusion strategy was either pPCI or PI. The main exposure under study was delayed reperfusion treatment, dichotomized into no or yes based on the recommended time limits. Hence, reperfusion was categorized as delayed if pPCI was not conducted within 120 min, or if fibrinolytic treatment was not administered within 30 min from FMC.^2,3^

The categorization of covariates was done as previously reported.^3^ Age was divided into four age groups: below 55, 55–64, 65–74, and 75–84 years. Based on diagnosis information from the NPR for the year prior to STEMI, comorbidity was grouped into none, medium (1–2 points), and high (≥3 points), corresponding to an adjusted Charlson comorbidity index [Patient Register Index (PRI)].^8^ Smoking status is registered in the NORMI, with the categories never smoker, ex-smoker (>1 month since the patient stopped smoking), and smoker. Time from symptom onset to FMC was grouped into ≤ 30, 31–120, and ≥ 121 min.

A tripartition of the original nine educational levels defined in the International Standard Classification of Education (ISCED) into lower, medium, and higher education was done according to the Norwegian Standard Classification of Education.^9^ Total after-tax income the year prior of the STEMI was divided into four approximately equally sized groups as described previously.^3^

### Statistical analyses

Data were analysed using SAS 9.4 (SAS Institute, Cary, NC, USA). Determinants that could potentially influence LVEF were identified based on findings from previous studies^3,10,11^ and assessed by directed acyclic graphs applying the 3.1 version of the tool available at Daggity.net.

We report baseline characteristics of patients as proportions (*n*) for categorical variables. For age, time from symptom onset to FMC, and after-tax income, even though categorized for analytical purposes, also median (Q1, Q3) is presented.

This study explores the relation between delayed reperfusion and the two outcome measures, mildly to moderately reduced LVEF (31–49%) and severely reduced LVEF (≤30%). The association between delayed reperfusion and mildly to moderately reduced LVEF (31–49%) was investigated examining risk ratio (RR) estimates derived from log-binomial regression.^12^ Due to convergence issues with log-binomial regression, we applied modified Poisson regression with robust standard errors to estimate RR for the association between delayed reperfusion and severely reduced LVEF (≤30%). Modified Poisson regression was chosen although the odds ratio estimates from logistic regression approximate the RR when the outcome risk is low, because when some of the subjects have higher risk than 10%, the odds ratio is known to be distorted away from the null value compared with the RR,^13^ and because modified Poisson regression composes conservative estimates, and hence reduces the risk of overestimating the association.^12^

Both outcomes were compared separately to normal LVEF (≥50%) in three models. The first model describes the unadjusted association between delayed reperfusion and the two outcome measures. As delayed reperfusion may occur after treatment following two different reperfusion strategies, the pPCI and the PI strategy, with different time recommendations, reperfusion strategy was included as the only covariate in the second model. In the third and full model, we additionally adjusted for age, gender, previous myocardial infarction (MI), smoking status, comorbidity, minutes from symptom onset to FMC, educational level, and income after tax.

### Ethical approval declarations

The study is based on secondary use of clinical and administrative register data. According to the Regional Committees for Medical and Health Research Ethics (REK), the study did not require approval by the REK (REK reference 2018/1955/REK nord). Exemption from the duty of confidentiality was granted by the REK for data from the NORMI and NPR and by Statistics Norway for their data. The data controller has carried out a data protection impact assessment. According to the Norwegian law, further ethical approval or obtaining informed consent was not required for this study. All methods were performed in accordance with the relevant guidelines and regulations. Public access to this type of data is closed, and data cannot be shared.

## Results

### Descriptive statistics

During 2015–2018, a total of 6567 Norwegian patients aged <85 years received reperfusion treatment for STEMI and were eligible for analysis. Among these patients, 57% had a normal LVEF (≥50%), while 39% had a mildly to moderately reduced LVEF (31–49%), and 4% had a severely reduced LVEF (≤ 30%), during the acute hospital admission (*Table 1*). A total of 43% (2825 out of 6567) of the patients with STEMI received delayed reperfusion.

**Table 1:** Left ventricular ejection fraction of patients with ST-segment elevation myocardial infarction in Norway 2015–2018, according to background patient characteristics (*n* = 6567)

	Normal LVEF (≥50%)	Reduced LVEF (31–49%)	Severely reduced LVEF (≤30%)	Total
Proportion of patients (*n*)	57.4% (3770)	38.5% (2531)	4.1% (266)	6567
Delayed reperfusion				
No	60.2% (2254)	36.8% (1377)	3.0% (111)	3742
Yes	53.7% (1516)	40.8% (1154)	5.5% (155)	2825
Reperfusion strategy				
pPCI	57.6% (3156)	38.5% (2112)	3.9% (214)	5482
Pharmacoinvasive strategy	56.6% (614)	38.6% (419)	4.8% (52)	1085
Age				
Median (Q1–Q3)	62 (54–69)	65 (56–72)	69 (61–76)	63 (55–71)
Below 55 years	65.0% (1018)	33.4% (522)	1.6% (25)	1565
55–64 years	59.5% (1192)	37.1% (743)	3.4% (69)	2004
65–74 years	54.2% (1078)	41.3% (822)	4.5% (90)	1990
75–84 years	47.8% (482)	44.0% (444)	8.1% (82)	1008
Gender				
Female	59.0% (861)	36.7% (536)	4.2% (62)	1459
Male	56.9% (2909)	39.1% (1995)	4.0% (204)	5108
Previous MI				
No	58.2% (3374)	38.0% (2204)	3.7% (215)	5793
Yes	51.2% (396)	42.2% (327)	6.6% (51)	774
Smoking status				
Never smoker	56.8% (928)	39.9% (652)	3.3% (54)	1634
Ex-smoker (>1 month)	55.8% (1165)	40.0% (835)	4.1% (86)	2086
Smoker	58.9% (1677)	36.7% (1044)	4.4% (126)	2847
Comorbidity				
No comorbidity	60.7% (3229)	35.9% (1908)	3.4% (180)	5317
Moderate comorbidity	46.4% (463)	48.0% (479)	5.6% (56)	998
High comorbidity	31.0% (78)	57.1% (144)	11.9% (30)	252
Minutes from symptom onset to FMC				
Median (Q1–Q3)	60 (30–128)	65 (30–145)	56 (23–142)	60 (30–135)
30 min or less	57.4% (1063)	37.4% (692)	5.2% (96)	1851
31–60 min	61.4% (930)	35.4% (536)	3.2% (48)	1514
61–120 min	56.5% (790)	40.0% (559)	3.5% (49)	1398
121 min or more	54.7% (987)	41.2% (744)	4.0% (73)	1804
Educational level				
Lower	55.4% (1102)	39.2% (781)	5.4% (107)	1990
Medium	57.3% (1894)	38.9% (1288)	3.8% (126)	3308
Higher	61.0% (774)	36.4% (462)	2.6% (33)	1269
Income after tax				
Median 1000 NOK (Q1–Q3)	321 (242–436)	304 (241–411)	276 (226–357)	311 (241–423)
Low	56.2% (909)	38.5% (622)	5.3% (86)	1617
Lower medium	52.5% (859)	42.6% (698)	4.9% (80)	1637
Higher medium	58.2% (954)	38.1% (624)	3.7% (60)	1638
High	62.6% (1048)	35.0% (587)	2.4% (40)	1675

The proportion of patients with mildly to moderately reduced LVEF was higher in patients who received delayed reperfusion (41%) compared with those who received timely reperfusion (37%). Also, for severely reduced LVEF, the proportion of patients was higher among those receiving delayed reperfusion (5.5%) compared with those receiving timely reperfusion (3%).

The proportion of patients with reduced LVEF was higher for patients in older age, among patients with previous MI and with comorbid conditions, and was lower with higher education and income, whereas time from symptom onset to FMC appeared to have a non-linear relationship with LVEF (*Table 1*).

## Results from regression models

### Associations between covariates and mildly to moderately reduced left ventricular ejection fraction (31–49%)

Patients receiving delayed reperfusion had 14% higher unadjusted risk of having a mildly to moderately reduced LVEF than patients receiving timely reperfusion treatment [RR 1.14, 95% confidence interval (CI) 1.07–1.21], Model 1 (*Table 2*). After adjustment for reperfusion strategy, the risk of a mildly to moderately reduced LVEF was 15% higher for patient receiving delayed reperfusion compared with patients receiving timely reperfusion (RR 1.15, 95% CI 1.06–1.25), Model 2. In the fully adjusted model, the risk of having mildly to moderately reduced LVEF after receiving delayed reperfusion was 11% higher relative to receiving timely reperfusion (RR 1.11, 95% CI 1.04–1.18), Model 3 (*Table 2*).

**Table 2:** Results from logistic regression for mildly to moderately reduced left ventricular ejection fraction in patients with ST-segment elevation myocardial infarction in Norway 2015–2018 (*n* = 6301)

	Model 1	Model 2	Model 3
	RR (95% CI)	RR (95% CI)	RR (95% CI)
Delayed reperfusion			
No	1.00	1.00	1.00
Yes	1.14 (1.07–1.21)	1.15 (1.06–1.25)	1.11 (1.04–1.18)
Reperfusion strategy			
pPCI		1.00	1.00
Pharmacoinvasive strategy		0.96 (0.86–1.07)	0.99 (0.91–1.08)
Age group			
Below 55 years			1.00
55–64 years			1.11 (1.02–1.22)
65–74 years			1.18 (1.08–1.29)
75–84 years			1.26 (1.14–1.40)
Gender			
Female			1.00
Male			1.12 (1.04–1.21)
Previous MI			
No			1.00
Yes			1.06 (0.97–1.15)
Smoking status			
Never smoker			1.00
Ex-smoker (>1 month)			0.96 (0.89–1.03)
Smoker			0.94 (0.87–1.02)
Comorbidity			
No comorbidity			1.00
Moderate comorbidity			1.31 (1.21–1.41)
High comorbidity			1.63 (1.48–1.81)
Minutes from symptom omset to FMC			
30 min or less			1.00
31–60 min			0.92 (0.84–1.00)
61–120 min			1.04 (0.95–1.13)
121 min or more			1.04 (0.97–1.13)
Educational level			
Lower			1.00
Medium			0.99 (0.92–1.06)
Higher			0.94 (0.86–1.03)
Income after tax			
Low			1.00
Lower medium			1.08 (0.99–1.17)
Higher medium			1.00 (0.92–1.10)
High			0.95 (0.86–1.04)

There was no difference in risk of a mildly to moderately reduced LVEF between patients receiving reperfusion treatment by the pPCI and the PI strategy. In the fully adjusted model, age, male gender, and comorbidity score were significantly associated with a mildly to moderately reduced LVEF, whereas a previous history of MI, time from symptom onset to FMC, educational level, and income were not, Model 3 (*Table 2*).

### Associations between covariates and severely reduced left ventricular ejection fraction (≤30%)

The risk of having a severely reduced LVEF was twice as high for patients receiving delayed reperfusion compared with patients receiving timely reperfusion (RR 1.98, 95% CI 1.56–2.50), unadjusted, Model 1 (*Table 3*). Adjusted for reperfusion strategy, the relative risk for patients with delayed reperfusion compared with patients with timely reperfusion was 2.00 (95% CI 1.56–2.57), Model 2 (*Table 3*). After further adjustments in the full Model 3, the relative risk of having a severely reduced LVEF for patients receiving delayed reperfusion was 1.76 (95% CI 1.37–2.25), indicating a 76% higher risk than for patients receiving timely reperfusion.

**Table 3:** Results from logistic regression for severely reduced left ventricular ejection fraction in patients with ST-segment elevation myocardial infarction in Norway 2015–2018 (*n* = 4036)

	Model 1	Model 2	Model 3
	RR (95% CI)	RR (95% CI)	RR (95% CI)
Delayed reperfusion			
No	1.00	1.00	1.00
Yes	1.98 (1.56–2.50)	2.00 (1.56–2.57)	1.76 (1.37–2.25)
Reperfusion strategy			
pPCI		1.00	1.00
Pharmacoinvasive strategy		0.94 (0.69–1.28)	1.07 (0.79–1.45)
Age group			
Below 55 years			1.00
55–64 years			2.33 (1.48–3.64)
65–74 years			3.00 (1.92–4.69)
75–84 years			5.31 (3.32–8.52)
Gender			
Female			1.00
Male			1.40 (1.06–1.85)
Previous MI			
No			1.00
Yes			1.38 (1.05–1.83)
Smoking status			
Never smoker			1.00
Ex-smoker (>1 month)			1.02 (0.73–1.41)
Smoker			1.43 (1.04–1.96)
Comorbidity			
No comorbidity			1.00
Moderate comorbidity			1.59 (1.18–2.13)
High comorbidity			3.61 (2.61–4.99)
Minutes from symptom onset to FMC			
30 min or less			1.00
31–60 min			0.56 (0.41–0.78)
61–120 min			0.63 (0.46–0.87)
121 min or more			0.66 (0.49–0.88)
Educational level			
Lower			1.00
Medium			0.79 (0.62–1.02)
Higher			0.61 (0.40–0.91)
Income after tax			
Low			1.00
Lower medium			1.01 (0.76–1.35)
Higher medium			0.95 (0.68–1.33)
High			0.74 (0.48–1.12)

The risk of a severely reduced LVEF was not significantly different between patients receiving reperfusion by the pPCI or the PI strategy. Increasing age, male gender, a previous history of MI, being a smoker, and comorbid conditions increased the risk of a severely reduced LVEF, while waiting more than 30 min from symptom onset to FMC and higher compared with lower education was associated with a lower risk.

## Discussion

### Principal finding

Delayed, compared with timely, reperfusion treatment in patients with STEMI increases the risk of a mildly to moderately reduced LVEF, and especially the risk of a severely reduced LVEF. No significant difference in the risk of reduced LVEF was observed between the pPCI strategy and the PI strategy.

### Time delay is a substantial risk factor for reduced left ventricular ejection fraction—independently of reperfusion strategy

The ESC recommendations concerning timely reperfusion for patients with STEMI is widely supported and based on the results of both RCT’s and observational studies.^1^

This national population-based cohort study confirms the importance of timely reperfusion for patients with STEMI. The adjusted risk of a mildly to moderately reduced LVEF was 11% higher, and the adjusted risk of a severely reduced LVEF was 76% higher for patients with STEMI receiving delayed reperfusion compared with patients with STEMI receiving timely reperfusion. A rather large proportion of patients received delayed reperfusion treatment during the same time period in Norway (44%), with substantial geographic variation due to long travel times to a PCI centre and few patients receiving pre-hospital fibrinolysis according to the PI strategy.^3^ Thus, this study points at an important area of quality improvement in Norwegian acute STEMI care.

There was no difference in the risk of reduced LVEF between patients receiving reperfusion by the pPCI strategy and the PI strategy, suggesting that timeliness is more important than reperfusion strategy. This is important because very few (307 out of 7607) patients with STEMI received timely reperfusion by the PI strategy in Norway during 2015–2018.^3^ Hence, expanded use of the PI strategy is the most promising approach to improve STEMI treatment in Norway, especially in distant and rural areas.

### Time from symptom onset to first medical contact

The extent of myocardial injury is strongly dependent on the duration of ischaemia before reperfusion;^14,15^ hence, we initially expected the risk of a severely reduced LVEF to increase by increasing time from symptom onset to FMC.

This was, however, not what we found. In the present study, patients waiting more than half an hour from symptom onset to FMC had a lower risk of a severely reduced LVEF, compared with patients waiting less than half an hour. This tendency has also been reported in other studies.^15,16^ Early presenters tend to be high-risk patients with large ST-segment elevations and high case fatality if not offered timely reperfusion treatment, while late presenters may have less severe ST-segment elevations.^16^ Patients with dramatic symptoms with a large part of the myocardium affected, and a higher risk of severe myocardial damage, may have a shorter delay from symptom onset to FMC. Hence, the results indicating a negative association between increasing time from symptom onset to FMC and severely reduced LVEF may be caused by differences in the severity of the myocardial infarction between early- and late-presenting patients.

The accuracy in the measurement of time from symptom onset to FMC may also have impact on these results. While the time of the FMC and the start of reperfusion treatment is registered fairly precise, the time of symptom onset is based on the patient’s recollection of when symptoms appeared, and may furthermore be influenced by non-specific symptoms, silent ischaemia, intermittent spontaneous reperfusion, collateral circulation, or prodromal angina.^17^

### Gender

Previous studies have found that women was associated with delayed reperfusion treatment^18,19^ and adverse outcomes.^20,21^ In a previous study based on the same data, we did not find any significant difference in the odds of receiving timely reperfusion between men and women,^3^ and in this study, we found that men had a significantly higher risk of both a mildly to moderately reduced and a severely reduced LVEF than women.

### Strengths and limitations

The present study is based on data from a national medical quality register, with nearly 90% completeness of patients for the study period. Due to the national unique personal identity number, we were able to supplement individual information on educational level, income, and patient administrative data from other national registries.

It is not possible to draw causal conclusions based on a cohort study with register data, due to the possibility of residual confounding after adjustments. Measurement, recall, and selection bias are other limitations. Also, the present study excluded patients older than 84 years of age, leaving out the oldest patients with STEMI. Some other patients were excluded because of missing information concerning outcome or covariates. This may not be at random. However, 80% (6567 out of 8255) of the patients with STEMI were included in the analysis, and there were no differences in mean comorbidity score and age of patients with timely and delayed reperfusion before and after excluding patients with missing information (results not shown).

This study is based on data from the NORMI that includes patients when they experience MI. Hence, we do not have pre-infarction measurements of LVEF for these patients. This is a limitation. However, patients with reported chronic heart failure were excluded from this study, and we adjust for previous MI in the full models.

Left ventricular ejection fraction is a useful, but imperfect, measure of heart failure. It can vary by imaging modality, haemodynamic loading conditions, heart rhythm, and on repeat measures even by the same modality for a single individual. Despite these limitations, decades of clinical trial evidence support this classification of heart failure,^22^ and reduced LVEF is a useful measure of an adverse outcome after acute STEMI treatment, supplementing mortality.

### Conclusions

Delayed reperfusion treatment is associated with 76% increased adjusted risk of severely reduced LVEF and is also associated with mildly to moderately reduced LVEF, compared with timely reperfusion. Reperfusion strategy, pPCI or PI, was not associated with reduced LVEF.

Further efforts to ensure timely reperfusion is crucial to avoid myocardial damage among patients with STEMI. Expanded use of the PI strategy should be considered as a measure to improve STEMI treatment in distant and rural areas.

## Lead author biography


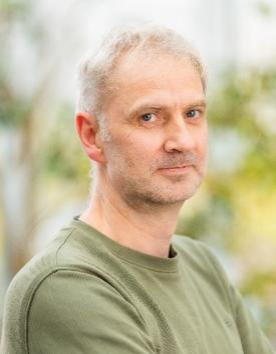


Bård Uleberg is a PhD student at UiT The Arctic University of Norway and an analyst at the North Norwegian Health Authority.

## Data Availability

The data underlying this article cannot be shared publicly due to restrictions. The original data were made available from the NORMI, the NPR, and Statistics Norway under license for the current study and with an exemption from the duty of confidentiality for involved researchers [granted by the Regional Committees for Medical and Health Research Ethics (REK) for data from NORMI and NPR, and by Statistics Norway for their data]. However, any researcher with approval of an exemption from professional secrecy requirements for the use of personal health data in research from the Regional Committee for Medical and Health Research Ethics (REK) would be able to create an almost identical (updated) data set by applying to the registries.
